# Supplementary material for: A Qualitative Exploration of the Functional, Social, and Emotional Impacts of the COVID-19 Pandemic on People Who Use Drugs
Source: Int J Environ Res Public Health. 2022 Aug 8;19(15):9751. doi: 10.3390/ijerph19159751 (PMC9367729; doi:10.3390/ijerph19159751)
Supplement: Supplementary file 1 [file ijerph-19-09751-s001.zip › ijerph-1827298-supplementary.pdf]

Supplemental Materials: Interview Guide

How have you been receiving your services during COVID?

Pathways-specific services (physical health and MAT), other non-Pathways services

How have you been impacted by COVID?

Housing: How has COVID-19 impacted your housing? Where are you staying right now? How is that different from before COVID-19 started?

Transportation: How are you getting around these days? Is this any different from before COVID-19 (easier or harder)?

Physically: How has your health been? How has it been impacted by COVID-19?

Financially: Have there been any changes to your benefits or your job(s)? How are you making money right now to meet your daily needs? Are you making money any differently than you did before?

Psychologically: How is your mental health these days? How has it been affected by COVID-19? Are you feeling anxious? Are you feeling lonely? Are you feeling isolated? Are you feeling irritable?

Food security: Do you have enough food in your house? Do you have enough food to eat? Do you run out of food or money for food toward the end of the month? Is this any different from before COVID? Do you think the quality of food is worse or better?

Who are the three people you are closest to right now?

For person 1 (etc.)

- What is their name? What is their relationship to you?
- How much are you seeing them in person now?
- How much are you talking to them on the phone now?
- How is this different from before COVID-19?
- What problems have you had connecting to this person?
- What's helped you to stay connected with this person?

How has your substance use been impacted by COVID?

Increase/decrease

How are you staying safe when you are using substances?

Access, contamination, cost, overdose concerns, FTS

Do you know anyone who has overdosed during this time? What happened?

How have you been protecting yourself against COVID-19?

What makes it easier to protect yourself?

What makes it harder?

Are you staying at home?

Are you wearing a mask?

Do you know anyone who has had COVID-19? What happened to them?

Have you been able to get tested? When, where and how?

How willing are you to get a vaccine?

How soon would you be willing to get it? If you could get vaccinated today, would you? Why or why not?

Is there anything about COVID-19 and your life that we should know about?
